# Temperature and microbial changes of corn silage during aerobic exposure

**DOI:** 10.5713/ajas.18.0566

**Published:** 2018-11-27

**Authors:** Seong Shin Lee, Hyuk Jun Lee, Dimas Hand Vidya Paradhipta, Young Ho Joo, Sang Bum Kim, Dong Hyeon Kim, Sam Churl Kim

**Affiliations:** 1 Division of Applied Life Science (BK21Plus, Institute of Agriculture & Life Sciences), Gyeongsang National University, Jinju 52828, Korea; 2 Faculty of Animal Science, Universitas Gadjah Mada, Yogyakarta, 55281, Indonesia; 3 Dairy Science Division, National Institute of Animal Science, RDA, Cheonan 31000, Korea; 4 Department of Animal Sciences, IFAS, University of Florida, Gainesville, FL 32608, USA

**Keywords:** Aerobic Stability, Corn Silage, Fermentation Indices, Inoculant, Silage Temperature

## Abstract

**Objective:**

This study was conducted to estimate the temperature and microbial changes of corn silages during aerobic exposure.

**Methods:**

Kwangpyeongok (KW) and Pioneer 1543 (PI) corn hybrids were harvested at 29.7% of dry matter and chopped to 3 to 5 cm lengths. Homo (*Lactobacillus plantarum*; LP) or hetero (*Lactobacillus buchneri*; LB) fermentative inoculants at 1.2×10^5^ colony forming unit/g of fresh forage was applied to the chopped corn forage which was then ensiled in quadruplicate with a 2×2 (hybrid×inoculant) treatment arrangement for 100 days. After the silo was opened, silage was sub-sampled for analysis of chemical compositions, *in vitro* digestibility, and fermentation indices. The fresh silage was continued to determine aerobic exposure qualities by recorded temperature and microbial changes.

**Results:**

The KW silages had higher (p<0.01) *in vitro* digestibilities of dry matter and neutral detergent fiber than those of PI silages. Silages applied with LB had higher (p<0.001) acetate concentration, but lower (p<0.01) lactate concentration and lactate to acetate ratio than those of LP silages. The interaction effect among hybrid and inoculant was detected in acetate production (p = 0.008), aerobic stability (p = 0.006), and lactic acid bacteria count (p = 0.048). The yeast was lower (p = 0.018) in LB silages than that in LP silages. During the aerobic exposure, PI silages showed higher (p<0.05) temperature and mold than KW silages, while LP silages had higher (p<0.05) lactic acid bacteria and yeast than LB silages.

**Conclusion:**

The results indicated that the changes of silage temperature during aerobic exposure seems mainly affected by mold growth, while applied LB only enhanced aerobic stability of PI silages.

## INTRODUCTION

Corn silage is a major source of forage for ruminants, which provides a higher energy level compared to other forages. Combining corn silage with protein sources has been applied to supply the nutritional requirement of beef or dairy cattle [1]. High concentration of water soluble carbohydrate (WSC) in corn forage causes higher potential of becoming overgrown by toxigenic fungi such as *Fusarium*, *Pencillium*, and *Aspergillus* genera [2,3] after fermentation compared to the other forages. Aerobic stability is an important factor to ensure corn silage quality and provide a good preserved nutrient with low amount of mold spores and toxicogenic compounds [4]. In addition, the undesirable spores have adverse effects on animal health and reduce their performance [5]. After opened the silo, yeast and mold will grow by degrading the nutrient and organic acids of silage that decrease aerobic stability [4]. The low aerobic stability decreases the utilization and nutritional value of corn silage [6].

In general, the aerobic stability of silage is usually calculated by the changes of temperature [7] or CO_2_ emission [8]. The temperature and CO_2_ levels of silage will increase during aerobic deterioration [4]. However, both these measurements do not always indicate clearly the growth of undesirable microorganisms during aerobic exposure. Consequently, the changes of temperature and microbes during aerobic exposure might be a direct indicator to determine the shelf life of corn silage.

Nowadays, varieties of hybrids have been developed to increase the productivity, quality, and efficiency of corn plants. In South Korea, Kwangpyeongok (KW) and Pioneer 1543 (PI) are the most widely planted corn hybrids in ruminant industry to supply feed forages. The KW hybrid is more adaptive resulting in higher dry matter (DM) yield and resistance to black-streaked dwarf virus [9], while PI hybrid produces higher grain yield [10]. The previous studies reported that application of hybrid had effect on fermentation quality and aerobic stability of corn silage [11–13]. On the other side, it is well known that applying a lactic acid bacteria (LAB) can increase fermentation and aerobic stability of silage. A homo fermentative LAB in silage enhances the fermentation quality by increasing lactate production [14], while a hetero fermentative LAB enhances the aerobic stability by increasing acetate production [15,16]. An interaction between hybrid and inoculant can be occurr and influence the silage quality [17].

Information of microbial growth such as LAB, yeast, and mold during aerobic exposure is limited regarding the silage temperature changes. In fact, this information could show the contribution of these microbes to an increase of silage temperature and facilitate an estimate shelf life of silage once the silo is opened. Therefore, this study was conducted to estimate aerobic stability of corn silage by observing temperature and microbial changes during aerobic exposure with two different hybrids and inoculants.

## MATERIALS AND METHODS

### Silage production

Two corn hybrids, KW (National Crop Experiment Station, Suwon, Korea) and PI (DuPont Pioneer, Aurelia, USA), were grown at the animal research unit, Gyeongsang National University, Jinju, Korea, and then harvested at 29.7% of DM. Both corn hybrids were chopped by conventional forage harvester (BHC-90, BUHEUNG Machinery Ltd., Jinju, Korea) into 3–5 cm lengths. After that, KW and PI forages were inoculated with bacterial inoculants: *Lactobacillus plantarum* (*L. plantarum*, LP; CMbio, Anseong, Korea) and *Lactobacillus buchneri* KACC12416 (*L. buchneri*, LB; Korean Culture Center of Microorganism, Seoul, Korea), respectively. Application rate of LP and LB were at 1.2×10^5^ cfu/g of fresh forage as the recommend level for silage inoculant [7]. All treatments were ensiled into 20 L mini silos (8 kg) in quadruplicate for 100 days. After ensiled for 100 days, a total 16 silos were opened, and the silages were sub-sampled for chemical compositions, fermentation indices, microbial counts, and aerobic stability.

### Chemical compositions

Forages and silages (500 g, respectively) were subsampled for chemical compositions, and dried at 55°C for 48 h, and ground to pass 1 mm screen of grinder (Cutting Mill, SHINMYUNG ELECTRIC Co., Ltd, Gimpo, Korea). The DM was determined in dry oven at 105°C for 24 h, and crude ash was measured with a muffle furnace at 550°C for 5 h. Crude protein (CP) and ether extract (EE) were determined by the producers of Kjeldahl using N analyzer (B-324, 412, 435 and 719 S Titrino, BUCHI, Essen, Germany) and Soxhlet [18], respectively. Neutral detergent fiber (NDF) and acid detergent fiber (ADF) were analysed by using Ankom^200^ fiber analyzer (Ankom Technology, Macedon, NY, USA) [19].

Animal care procedure for cannulated Hanwoo heifers in the present study was approved by animal ethical committee of Gyeongsang National University, Jinju, South Korea. Rumen fluid was collected before morning feeding from two cannulated Hanwoo heifers fed rice straw and commercial concentrate at 8:2 ratio. The collected rumen fluid was composited and filtered via 2 layers of cheesecloth. Forage and silage samples were incubated in mixture of rumen fluid and buffer solution at 1:2 ratio to determine *in vitro* digestibility of DM (IVDMD) and NDF (IVNDFD) through an Ankom Daisy (Ankom Technology, USA) [20].

### Fermentation indices

Twenty grams of silage were mixed along with 200 mL of distilled water to make silage extraction for pH, ammonia-N, lactate, volatile fatty acid (VFA), and microbial counts. The pH was measured with electric pH meter (SevenEasy, Mettler Toledo, Greifensee, Switzerland) and ammonia-N was analyzed by colorimetry [21]. For lactate and VFA analyses, the silage extraction was centrifuged at 5,645×*g* for 15 min and collected the supernatant. The concentrations of lactate and VFA were determined using HPLC (L-2200, Hitachi, Tokyo, Japan) fitted with a UV detector (L-2400; Hitachi, Japan) and a column (Metacarb 87H; Varian, Palo Alto, CA, USA) described by Muck and Dickerson method [22].

### Microbial counts and aerobic stability

Fresh silage extraction (first dilution) was continued in several dilutions (10^−5^ to 10^−7^) to determine LAB, yeast, and mold counts. The lactobacilli MRS agar medium (MRS; Difco, Detroit, MI, USA) was used for the isolation and count of LAB, and potato dextrose agar medium (PDA; Difco, USA) was used for the isolation and counts of yeast and mold. The lactobacilli MRS agar medium was placed in a CO_2_ incubator (Thermo Scientific, Waltham, MA, USA) at 30°C for 48 h and PDA medium was incubated at 28°C for 72 h in aerobic incubator (Johnsam Corporation, Boocheon, Korea). Visible colonies were counted from the agar medium at appropriate dilutions and the number of colony forming units (cfu) was expressed per gram of silage. The aerobic stability was determined by transferring silage (2 kg) into open-top polyethylene containers. Three thermocouple wires were placed to the center of silage and connected to data loggers (TR-60CH, MORGAN, Hong Kong, China) along with a computer that recorded temperature at every 30 min for 30 h. The silage containers were covered with 2 layers of cheesecloth to prevent drying and contamination by dust. The aerobic stability was measured by the time required to raise the silage temperature 2°C above the ambient temperature (20°C) as suggested by previous study [7]. During 8 days of aerobic exposure, silage from polyethylene containers was sub-sampled (100 g, respectively) in every day to determine the microbial counts. The protocol of microbial counts was same as described before.

### Statistical analysis

This experiment had a completely randomized design with a 2 (hybrid; KW vs PI)×2 (inoculant; LP vs LB) factorial arrangement of treatments. All data of chemical compositions, fermentation indices, aerobic stability, microbe counts, and temperature of corn silages were analyzed using general linear model procedure of Statistical Analysis System ver. 9.3 [23] and a model containing hybrid, inoculant, and interactions of these terms. Significance difference was declared at p<0.05.

## RESULTS

### Chemical composition of corn forage and silage

The concentrations of DM, CP, NDF, ADF, IVDMD, and IVNDFD of KW forage before ensiling were 29.7%, 9.65%, 51.7%, 28.1%, 60.3%, and 44.9%, respectively (Table 1). Whereas, the concentrations of DM, CP, NDF, ADF, IVDMD, and IVNDFD of PI forage before ensiling were 29.7%, 10.4%, 47.0%, 23.5%, 60.6%, and 45.2%, respectively.

After silo opened, KW silages had higher concentrations of DM (p = 0.016; 28.4% vs 26.7%), EE (p = 0.030; 3.86% vs 3.78%), crude ash (p<0.001; 6.85 vs 5.96), IVDMD (p<0.001; 71.4% vs 64.3%), and IVNDFD (p = 0.001; 50.2% vs 45.2%) than PI silages (Table 2). However, PI silages had higher concentrations of CP (p<0.001; 9.61% vs 8.62%) and ADF (p = 0.009; 27.1% vs 25.9%) than KW silages. The NDF concentration was not affected (p>0.05) by corn hybrids. However, LP silages had higher CP (p = 0.039; 9.24% vs 8.99%), but lower EE concentrations (p<0.001; 3.75% vs 3.89%) than LB silages. The interactions between hybrid and inoculant only affected EE (p = 0.002) and NDF (p = 0.009) concentrations.

### Fermentation indices of corn silage

The KW silages had lower concentrations of ammonia-N (p = 0.001; 0.09% vs 0.11%) and acetate (p = 0.025; 0.88% vs 1.00%) than PI silages (Table 3). LP silages had higher lactate concentration (p = 0.002; 3.12% vs 2.07%) and lactate to acetate ratio (p = 0.002; 4.18 vs 2.11), but lower acetate concentration (p< 0.001; 0.76% vs 1.12%) than LB silages. The interaction between hybrid and inoculant only affected (p = 0.008) acetate concentration.

### Aerobic stability and microbial count of corn silage

Aerobic stability of corn silage was higher in KW than in PI silages (p<0.001; 29.2 vs 13.4), but not affected (p>0.05) by inoculant (Table 4). The interaction effect among hybrid and inoculant was detected in aerobic stability (p = 0.006). Silages inoculated with LP had higher LAB count (p = 0.002; 6.15 vs 4.50 log10 cfu/g) and yeast count (p = 0.018; 6.08 vs 5.21 log10 cfu/g) than LB. The mold count was not detected in all silages. The interaction between hybrid and inoculant affected (p = 0.016) aerobic stability and LAB (p = 0.048).

### Temperature change of corn silage during aerobic exposure

**Figure 1:**
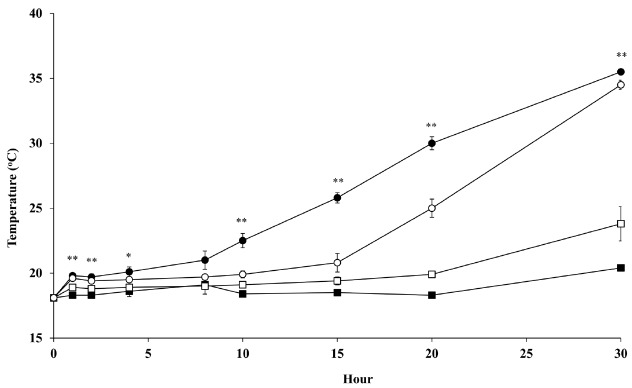
Effects of inoculant application on temperature of corn silage during aerobic exposure. Kwangpyeongok corn silage inoculated with *Lactobacillus plantarum* (■) or *Lactobacillus buchneri* (□); Pioneer 1543 corn silage inoculated with *Lactobacillus plantarum* (●) or *Lactobacillus buchneri* (○). The respective significance levels of hybrid, inoculant, hybrid×inoculant, and SEM for temperature are p<0.001, p = 0.087, p = 0.319, and SEM = 3.783. Values differ among groups within same hour * p<0.05 and ** p<0.01. SEM, standard error of mean.

A change of temperature in corn silages during aerobic exposure is shown in Figure 1. The KW silages produced lower (p<0.05) temperature than PI silages during the observation. However, it was not affected (p>0.05) by inoculant. The temperatures of PI silage applied LP inoculant were highest (p< 0.05) at 1, 2, 4, 8, 10, 15, 20, and 30 h of aerobic exposure compared to the other silages. The time when silage temperature was 2°C higher than the ambient temperature, were in the order of PI silage with LP inoculant (10.3 h), PI silage with LB inoculant (15.0 h), KW silage with LB inoculant (21.9 h), and KW silage with LP inoculant (29.5 h).

### Microbial change of corn silage during aerobic exposure

**Figure 2:**
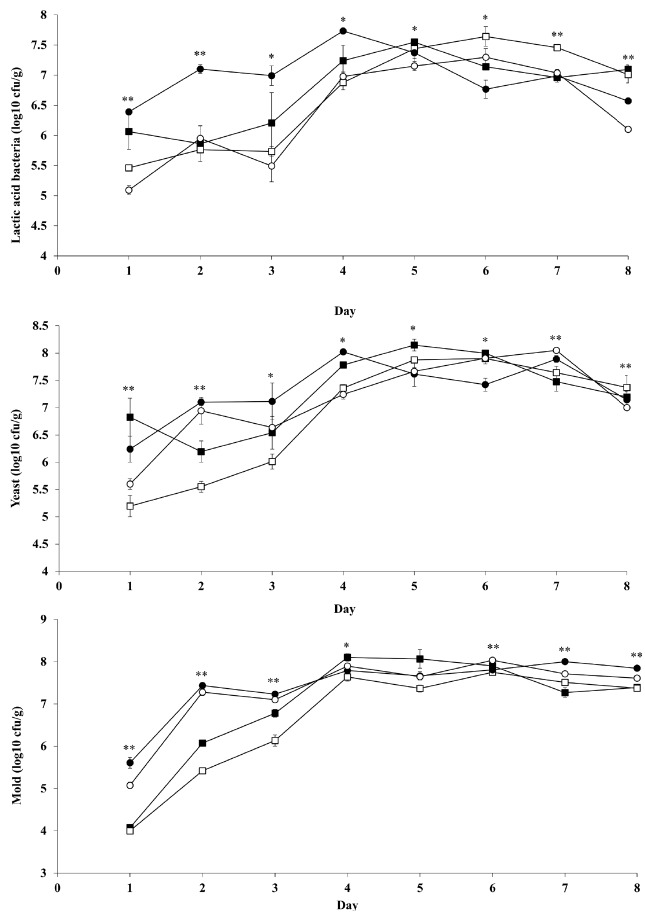
Effects of inoculant application on lactic acid bacteria, yeast, and mold counts of corn silages during aerobic exposure. Kwangpyeongok corn silage inoculated with *Lactobacillus plantarum* (■) or *Lactobacillus buchneri* (□); Pioneer 1543 corn silage inoculated with *Lactobacillus plantarum* (●) or *Lactobacillus buchneri* (○). The respective significance levels of hybrid, inoculant, hybrid×inoculant, and SEM for lactic acid bacteria, yeast, and mold are p = 0.856, p = 0.039, p = 0.126, and SEM = 0.809; p = 0.388, p = 0.099, p = 0.842, and SEM = 0.879; p = 0.063, p = 0.493, p = 0.598, and SEM = 1.072, respectively. Values differ between groups within same hour * p<0.05 and ** p<0.01. SEM, standard error of mean.

The counts of LAB, yeast, and mold in corn silage during aerobic exposure are presented in Figure 2. The growth of microbes was not affected (p>0.05) by corn hybrid and inoculant, except LAB that was higher in LP than in LB inoculant applications (p = 0.039). The LAB, yeast, and mold counts were gradually increased by 4 days of aerobic exposure, after then stable by 8 days. Among the treatments, the LAB count was highest (p<0.05) in PI silage with LP inoculant by 4 days of aerobic exposure. The yeast count was lowest (p<0.05) in KW silage with LB inoculant by 3 days of aerobic exposure, and in both hybrid silages with LB inoculant at 4 days. The mold count was lowest (p<0.05) in KW silage with both inoculants at 1 day, and in KW silage with LB inoculant at 2 and 3 days.

## DISCUSSION

The chemical compositions of KW and PI hybrids were in the expected range for corn grown in South Korea [9,10,24,25]. In general, the differences of silage chemical compositions among hybrids are caused by those of forages [12,13]. In the present study, the results of higher EE and crude ash concentrations in KW silage, but lower CP and ADF concentrations than PI silages might be due to those compositions of corn forages. On the other side, a higher CP concentration by LP than LB silages indicated lower proteolysis in homo fermentative LAB [26]. The reason for higher EE concentration in LP than LB silages was not clear, but it could be due to higher total organic acid concentration in LP silages, which caused a lower nutrient loss, including EE concentration during fermentation [6]. The NDF concentration was affected by interaction among hybrid and inoculant that presented higher concentration in PI silage, but lower in KW silage when using LB inoculant in both hybrids. The effects of inoculant on chemical compositions of silage can vary depending hybrids [27]. The higher IVDMD and IVNDFD of KW than PI silages could be influenced by lower ADF concentration in KW compared to PI silages [28].

In fermentation indices, pH of all silages was below 4.0 which could be an indicator of enough acidification to inhibit the growth of undesirable microbes [7,15,27]. In the present study, the absence of butyrate and mold in all silages might be caused by rapid acidification and low pH in silage (Tables 3, 4). Ammonia-N in silage is a by-product of CP degradation during ensiling and its concentration could be affected by CP concentration of forages [6,27]. The higher ammonia-N in PI than in KW silages might be caused by the higher CP concentration in PI than in KW corn forages (10.40% vs 9.65%). The differences of chemical composition among KW and PI forages caused LB inoculant to produce a different response in producing organic acids. According to McDonald et al [6], the WSC concentration and buffer capacity of forage influences the LAB development during ensiling. The previous studies also reported that organic acid profiles of silage could be influenced by interaction effect between hybrid and inoculant that also caused variation in results depending on types of inoculant and hybrid [17,27]. In the present study, LP as homo fermentative inoculant increased lactate concentration in both hybrids, whereas LB as hetero fermentative inoculant increased acetate concentration more effectively in PI silage than in KW silage (hybrid and inoculant interaction). These results of organic acid concentrations supported lactate to acetate ratio in the present study, which was higher ratio in LP and KW silages than in LB and PI silages, respectively.

According to previous study [14], inoculation of silage with homo fermentative LAB had beneficial effects in promoting the LAB growth, which was similar result with LAB count in the present study. Applied LB inoculant in the present study decreased the growth of yeast more effectively than LP due to higher acetate production (Table 3). Acetate has a role as antimicrobial compound against the undesirable microbes [29].

Application of hetero fermentative LAB increased aerobic stability of silage because it increases acetate concentration during ensiling [15,16,29]. In the present study, applied LB inoculant only increased aerobic stability in PI silage without any improvement in KW silage, which was similar to results in a previous study [13]. In aerobic stability of KW silages, the reason for no effect among inoculants could be supported partially by the similar acetate concentrations of LP and LB inoculants (0.78% vs 0.98%). Temperatures of all silages were gradually increased during the aerobic exposure under room temperature (20°C), and KW silages had 2 times higher aerobic stability than PI silages. This might be caused by the hybrid effect [13]. Even though PI silages had slightly higher acetate concentration (1.00% vs 0.88%) than KW silages, it showed higher population of mold by 3 days of aerobic exposure (Figure 2). This might contribute to a decrease aerobic stability. The shelf life of silage is usually estimated by aerobic stability [6]. Determination of aerobic stability should consider not only a change of temperature but also microbial growth during aerobic conditions. In the present study, LAB count after silo opened was influenced by interaction effect among hybrid and inoculant that agreed with previous study [27]. And during aerobic exposure, LAB still could grow 4–5 days because several strains were classified as aerotolerant anaerobes, such as *L. plantarum*, *Leuconostoc mesenteriodes*, and *Streptococcus lactis* [30]. Also, anaerobic condition in the bottom of silage container still allowed LAB to grow. This principle could be applied to bunker silo that represented the front side of silo in aerobic condition, but the end side of silo in anaerobic condition [7]. The growth of LAB did not inhibit yeast and mold after silo open in the present study. In yeast count, KW silage with LP inoculant showed the similar count at day 0 (6.15 log10 cfu/g) and 2 (6.19 log10 cfu/g) of aerobic exposure. Moreover, KW silage with LB inoculant still presented the yeast count at 5.5 log10 cfu/g during 2 days of aerobic exposure. It indicated that yeast count in KW silages with both inoculants was relatively stable or slow growth for 2 days, even though its temperature was 2°C higher than ambient. LP and LB inoculants presented a similar effect on yeast growth in KW silage due to both inoculants having a similar concentration of acetate (Table 3). In PI silages, both inoculant treatments showed a similar count at day 0 (5.63 log10 cfu/g) and 1 (5.91 log10 cfu/g) of aerobic exposure. It also indicated the yeast count in PI silage was relatively stable or slow growth until day 1, even though the temperature was reported 16°C higher than ambient. The growth of yeast stimulated the mold that might have direct effect on increases of temperature in the present study. In 1 day of aerobic exposure, mold was detected and generally the aerobic stability finished in all treatments. This might indicate that mold was the main contributor to increases of silage temperature in the present study. Previous study reported that yeast and mold utilized remaining nutrients such as lactate and WSC to grow during aerobic exposure and decrease aerobic stability of silage as a response of temperature increases [15,29]. However, the present study indicated that increase of silage temperature was mainly influenced by mold, while the yeast contribution was to stimulate the mold growth. A high acetate concentration in LB silages resulted in a lower yeast count than LP silages, which was similar to a previous study [15].

## CONCLUSION

During aerobic exposure, LAB could still grow for 4 to 5 days of aerobic condition, but it did not inhibit the growth of undesirable microbes. The yeast stimulated the mold growth in the present study. Changes of silage temperature during aerobic exposure seems mainly due to mold growth. Inoculation of silages with LP as homo fermentative inoculant improved the fermentation indices in both hybrids, while inoculation with LB as hetero fermentative inoculant only enhanced aerobic stability of PI hybrid.

## Figures and Tables

**Table 1:** Chemical compositions and *in vitro* digestibility of corn forages before ensiling (%, dry matter)

Items	KW	PI
Dry matter	29.7	29.7
Crude protein	9.65	10.4
Ether extract	3.98	3.50
Crude ash	6.10	5.87
Neutral detergent fiber	51.7	47.0
Acid detergent fiber	28.1	23.5
*In vitro* dry matter digestibility	60.3	60.6
*In vitro* neutral detergent fiber digestibility	44.9	45.2

KW, Kwangpyeongok hybrid; PI, Pioneer 1543 hybrid.

**Table 2:** Effects of inoculant application on chemical composition and *in vitro* digestibility of corn silages ensiled for 100 days (%, DM)

Items	KW1)		PI1)		SEM	Contrast2)		
		
	LP	LB	LP	LB		HY	INO	HY×INO
Dry matter	28.3	28.5	26.3	27.1	0.948	0.016	0.406	0.570
Crude protein	8.81	8.43	9.67	9.55	0.175	<0.001	0.039	0.228
Ether extract	3.80	3.91	3.69	3.86	0.030	0.030	<0.001	0.002
Crude ash	6.94	6.75	5.93	5.98	0.104	<0.001	0.304	0.164
NDF	47.4	46.5	46.5	48.0	0.555	0.499	0.379	0.009
ADF	25.9	25.8	26.5	27.6	0.787	0.009	0.151	0.112
IVDMD	71.4	71.3	64.3	64.2	0.665	<0.001	0.842	1.000
IVNDFD	50.2	50.1	45.4	44.9	0.716	0.001	0.585	0.647

DM, dry matter; SEM, standard error of mean; NDF, neutral detergent fiber; ADF, acid detergent fiber; IVDMD, *in vitro* DM digestibility; IVNDFD, *in vitro* NDF digestibility.

1) KW, Kwangpyeongok hybrid; PI, Pioneer 1543 hybrid; LP, corn silage inoculated with *Lactobacillus plantarum*; LB, corn silage inoculated with *Lactobacillus buchneri*.

2) HY, hybrid effect; INO, inoculant effect; HY×INO, interaction between hybrid and inoculant.

**Table 3:** Effects of inoculant application on fermentation indices of corn silages ensiled for 100 days

Items	KW1)		PI1)		SEM	Contrast2)		
		
	LP	LB	LP	LB		HY	INO	HY×INO
pH	3.85	3.88	3.88	3.93	0.075	0.331	0.404	0.853
Ammonia-N (%)	0.09	0.08	0.11	0.11	0.008	0.001	0.440	0.953
Lactate (%)	3.14	2.13	3.09	2.01	0.213	0.623	0.002	0.828
Acetate (%)	0.78	0.98	0.74	1.25	0.062	0.025	<0.001	0.008
Propionate (%)	0.15	0.14	0.15	0.19	0.033	0.371	0.479	0.244
Butyrate (%)	ND	ND	ND	ND	-	-	-	-
La:Ac	4.03	2.17	4.18	1.61	0.319	0.227	0.002	0.353

SEM, standard error of mean; La:Ac, lactate to acetate ratio; ND, not detected.

1) KW, Kwangpyeongok hybrid; PI, Pioneer 1543 hybrid; LP, corn silage inoculated with *Lactobacillus plantarum*; LB, corn silage inoculated with *Lactobacillus buchneri*.

2) HY, hybrid effect; INO, inoculant effect; HY×INO, interaction between hybrid and inoculant.

**Table 4:** Effects of inoculant application on aerobic stability and microbial counts of corn silages ensiled for 100 days

Items	KW1)		PI1)		SEM	Contrast2)		
		
	LP	LB	LP	LB		HY	INO	HY×INO
Aerobic stability (h)	32.8	25.5	8.08	18.7	3.302	<0.001	0.468	0.006
LAB (log10 cfu/g)	6.04	5.00	6.25	4.00	0.301	0.136	0.002	0.048
Yeast (log10 cfu/g)	6.15	5.17	6.01	5.25	0.327	0.987	0.018	0.987
Mold (log10 cfu/g)	ND	ND	ND	ND	-	-	-	-

LAB, lactic acid bacteria; SEM, standard error of mean; cfu, colony forming unit; ND, not detected.

1) KW, Kwangpyeongok hybrid; PI, Pioneer 1543 hybrid; LP, corn silage inoculated with *Lactobacillus plantarum*; LB, corn silage inoculated with *Lactobacillus buchneri*.

2) HY, hybrid effect; INO, inoculant effect; HY×INO, interaction between hybrid and inoculant.
